# AlbEmo: A perceptually validated emotional speech dataset for Albanian

**DOI:** 10.1016/j.dib.2026.113282

**Published:** 2026-09-23

**Authors:** Emiranda Loka, Ilma Lili, Alda Kika, Ana Ktona, Linda Mëniku, Alex Thomo

**Affiliations:** a University of Tirana, Faculty of Natural Sciences, Department of Informatics, Bulevardi Zogu I, Nr. 25/1, Tirana, Albania; b University of Tirana, Faculty of History and Philology, Department of Linguistics, Rruga e Elbasanit, Tirana, Albania; c University of Victoria, Department of Computer Science, PO Box 3055 STN CSC, Victoria, BC V8W 3P6, Canada

**Keywords:** Emotion recognition, Affective computing, Acted emotions, Paralinguistics, Low-resource language, Balkan languages, Inter-rater agreement

## Abstract

AlbEmo is, to our knowledge, the first emotional speech corpus for Albanian, a language covered by no current multilingual speech-emotion benchmark. Ten native Albanian speakers each produced six semantically neutral sentences in seven acted emotional styles (Ekman’s six basic emotions, anger, disgust, fear, happiness, sadness, and surprise, plus a neutral reference), yielding 420 single-utterance recordings (48 kHz, 16-bit, mono WAV). Each speaker recorded alone through a purpose-built offline browser tool with a cardioid USB microphone, one utterance at a time. The emotional content was validated with a forced-choice perceptual listening test in which 1260 native-listener judgements give an overall recognition rate of 73% against a chance level of 14%, and Fleiss’ κ=0.59, in the range reported for comparable acted corpora. As a reference point for machine use, a baseline built on frozen self-supervised speech embeddings (WavLM) separates the seven emotions at 74% accuracy per speaker. The corpus and the anonymized listening-test responses are released openly under CC-BY-4.0, extending speech-emotion resources to an under-resourced Balkan language.

Specifications Table

**Table:** 

Subject	Computer Sciences
Specific subject area	Speech emotion recognition; affective computing; acted emotional-speech corpora for an under-resourced language (Albanian).
Type of data	Raw and analyzed. Audio: uncompressed WAV, 48 kHz, 16-bit, mono. Tables; figures; CSV (per-response perceptual-test data); JSON (corpus metadata).
Data collection	Ten native Albanian speakers (five female, five male) self-recorded at the University of Tirana in July 2026, one utterance at a time, through a purpose-built offline browser tool. Microphone: Blue Yeti (cardioid) at ∼15–20 cm; browser audio processing (echo cancellation, noise suppression, automatic gain) disabled. Each produced six semantically neutral carrier sentences (three short, three long) in a fixed sequence of seven acted emotional styles (neutral plus Ekman’s six basic emotions), giving 42 utterances per speaker and 420 in total. Labels were then validated in a forced-choice listening test by four native-Albanian listeners, three ratings per clip.
Data source location	Institution: University of Tirana. City/Town/Region: Tiranë. Country: Albania.
Data accessibility	Repository name: Zenodo. Data identification number: 10.5281/zenodo.21638085. Direct URL to data: https://doi.org/10.5281/zenodo.21638085. Instructions for accessing these data: freely available under a Creative Commons Attribution 4.0 International licence, no registration or request required. The deposit holds the audio, the metadata, and the perceptual-test responses; the analysis scripts and the browser tools are in a public code repository, https://github.com/thomouvic/albemo-public.
Related research article	None.

## 1 Value of the Data

• AlbEmo is, to our knowledge, the *first* emotional speech corpus for Albanian, a language covered by no current multilingual speech-emotion benchmark (e.g. EmoBox [1]). It closes a concrete gap in language coverage.
• The corpus follows the design of established acted-emotion corpora (EmoDB, RAVDESS, SUBESCO), using an Ekman-based emotion set, identical to SUBESCO’s, on fixed, semantically neutral sentences, so that the emotional signal lies in vocal delivery. Its 420 utterances form a complete speaker × emotion × sentence grid (10×7×6), so every cell is filled and the design is balanced by construction, making AlbEmo directly comparable and usable for cross-lingual and cross-corpus speech-emotion research.
• Every emotion label is perceptually validated by 1260 native-listener judgements, three independent ratings per clip. The released per-response CSVs carry each individual judgement, so users can weight or filter training examples by listener agreement instead of treating every label as equally certain.
• The corpus serves speech-technology researchers building or benchmarking multilingual and cross-lingual emotion recognition, for whom it adds a language absent from current pretraining benchmarks; phoneticians and linguists studying Albanian prosody; and transfer-learning and low-resource research needing a fully labelled target language outside the usual English and German resources.
• Everything needed to reuse or reproduce the corpus is released under CC-BY-4.0. The archived deposit holds the audio, the metadata, and the anonymized listening-test responses; the analysis scripts and the two offline browser tools used to build it (Fig. 4), in Albanian and English, are kept in a public code repository, so that they can be corrected without altering an archived record. The tools need no server and no installation, so the same protocol can be applied to another under-resourced language.

## 2 Background

Albanian is spoken by roughly seven million people but, to our knowledge, has no openly available emotional-speech dataset and no presence in multilingual speech-emotion benchmarks; a search of Zenodo, Hugging Face, the ELRA/LDC catalogues, and the literature returned none. How far emotion models transfer to Albanian therefore cannot be tested at all. We compiled AlbEmo to close that gap with a resource directly comparable to the datasets the field already uses.

The design follows the acted-corpus tradition established by EmoDB, RAVDESS and SUBESCO (Table 1), using a fixed set of semantically neutral carrier sentences spoken in a common set of emotional styles, so that the emotion is carried mainly by the voice rather than the words. The emotion set is Ekman’s six basic emotions plus a neutral reference [2,3], identical to SUBESCO’s, which keeps cross-corpus comparison straightforward.

**Table 1:** AlbEmo in the family of acted emotional-speech corpora it is modelled on. AlbEmo's emotion set matches SUBESCO exactly; RAVDESS additionally includes calm, and EmoDB has boredom in place of surprise. IEMOCAP elicited five emotions (happiness, anger, sadness, frustration, neutral) but annotated nine, with disgust and fear rare.

Corpus	Language	Emotions	#Speakers	#Sentences
EmoDB [4]	German	7 (six basic less surprise; + boredom, neutral)	10	10
RAVDESS [5]	English	8 (six basic + neutral, calm)	24	2
SUBESCO [6]	Bangla	7 (Ekman’s six + neutral)	20	10
IEMOCAP [7]	English	5 elicited, 9 annotated (conversational)	10	dialogue
**AlbEmo (this work)**	Albanian	7 (Ekman’s six + neutral)	10	6

Two methodological commitments shaped the collection. First, the labels are not taken on trust from the performers. Every clip was validated in a forced-choice perceptual listening test by native listeners, and every individual judgement is released, so users can weight or filter examples by listener agreement instead of treating every label as equally certain. Second, the full speaker × emotion × sentence grid is filled, so the dataset is balanced by construction rather than after the fact.

## 3 Data Description

AlbEmo is a dataset of 420 emotional speech recordings in Albanian [8], produced by ten native speakers who each spoke six semantically neutral sentences in seven emotional styles, with every emotion label validated by a native-listener perceptual test.

### 3.1 Contents and organization

The corpus contains 420 audio recordings, one for every combination of 10 speakers, 7 emotional styles, and 6 sentences. Each recording is a single spoken sentence, on average 3.15 s long (range 1.2–10.1 s), stored as uncompressed WAV (48 kHz, 16-bit, mono), 22.1 min and 127 MB in total. Files are organized one directory per speaker, then one sub-directory per emotion: recordings/sp*NN*/*emotion*/sentence_*K*.wav where sp*NN* is the anonymized speaker (NN=01…10), *emotion* is the Albanian emotion marker (Table 2), and K=1…6 indexes the sentence (Table 3). For example, recordings/sp02/trishtim/sentence_4.wav is speaker 2 producing sentence S04 in a sad voice.

**Table 2:** The seven emotional styles, in recorded order, with the Albanian marker used as the folder name and the internal code.

Order	Albanian marker	English	Code
1	Neutral	neutral	e07
2	*G*ëzim	happiness	e04
3	Trishtim	sadness	e05
4	Zemërim	anger	e01
5	Frikë	fear	e03
6	Habi	surprise	e06
7	Neveri	disgust	e02

**Table 3:** The six semantically neutral carrier sentences (three short, three long) with an English gloss.

Code	Albanian	English gloss
S01	U hap dera.	The door opened.
S02	Makina është në garazh.	The car is in the garage.
S03	Ajo po flet në telefon.	She is talking on the phone.
S04	Letra erdhi dje në mëngjes në adresën time.	The letter came to my address yesterday morning.
S05	Dritaret e shtëpisë shohin nga rrugica e vjetër.	The windows of the house look onto the old alley.
S06	Shiu filloi në orën pesë dhe ra gjithë natën.	The rain started at five and fell all night.

The seven emotional styles are the six basic emotions of Ekman [2,3] plus a neutral reference (Table 2). The six carrier sentences (Table 3) are semantically neutral, three short and three long.

### 3.2 Acoustic overview

Fig. 1 shows the same sentence spoken in all seven emotions by one speaker, where the fixed words yield visibly different pitch, harmonic structure, and tempo. Clip duration itself varies with emotion (Fig. 2); sad utterances are the longest (mean 3.7 s) and fearful the shortest (mean 2.8 s), consistent with the slower tempo of sadness and the clipped delivery of fear. Pitch and intensity also vary with emotion (Table 4). Happiness and fear are highest in mean F0, anger and happiness are the loudest, and sadness is both the lowest in pitch and the softest.

**Fig. 1:**
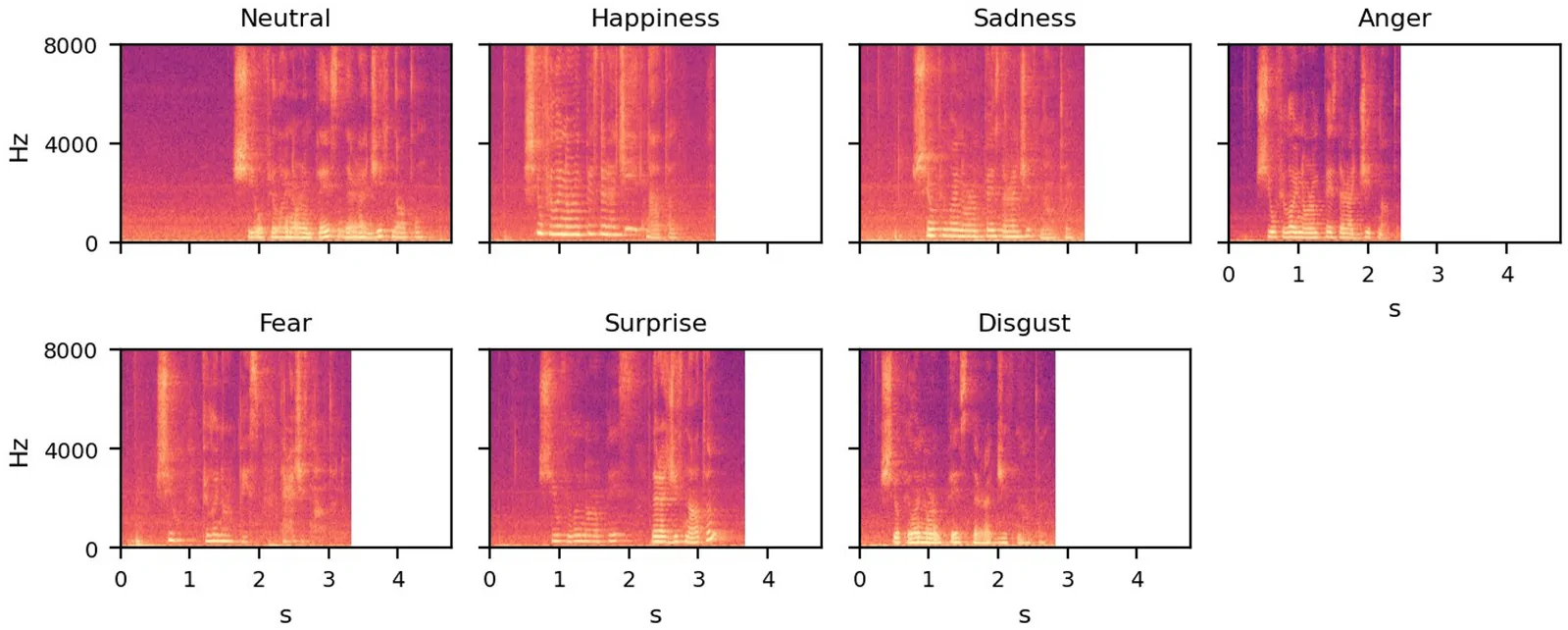
The same carrier sentence (S06, speaker sp02) produced in all seven emotional styles. Words are identical; pitch, harmonic detail, and duration change with the intended emotion. Spectrograms use a 1024-point FFT with 50% overlap and are displayed to 8 kHz. Both axes are shared across panels, so blank space at the right of a panel means the utterance ended earlier. Here the neutral take runs 4.8 s and the angry one 2.5 s.

**Fig. 2:**
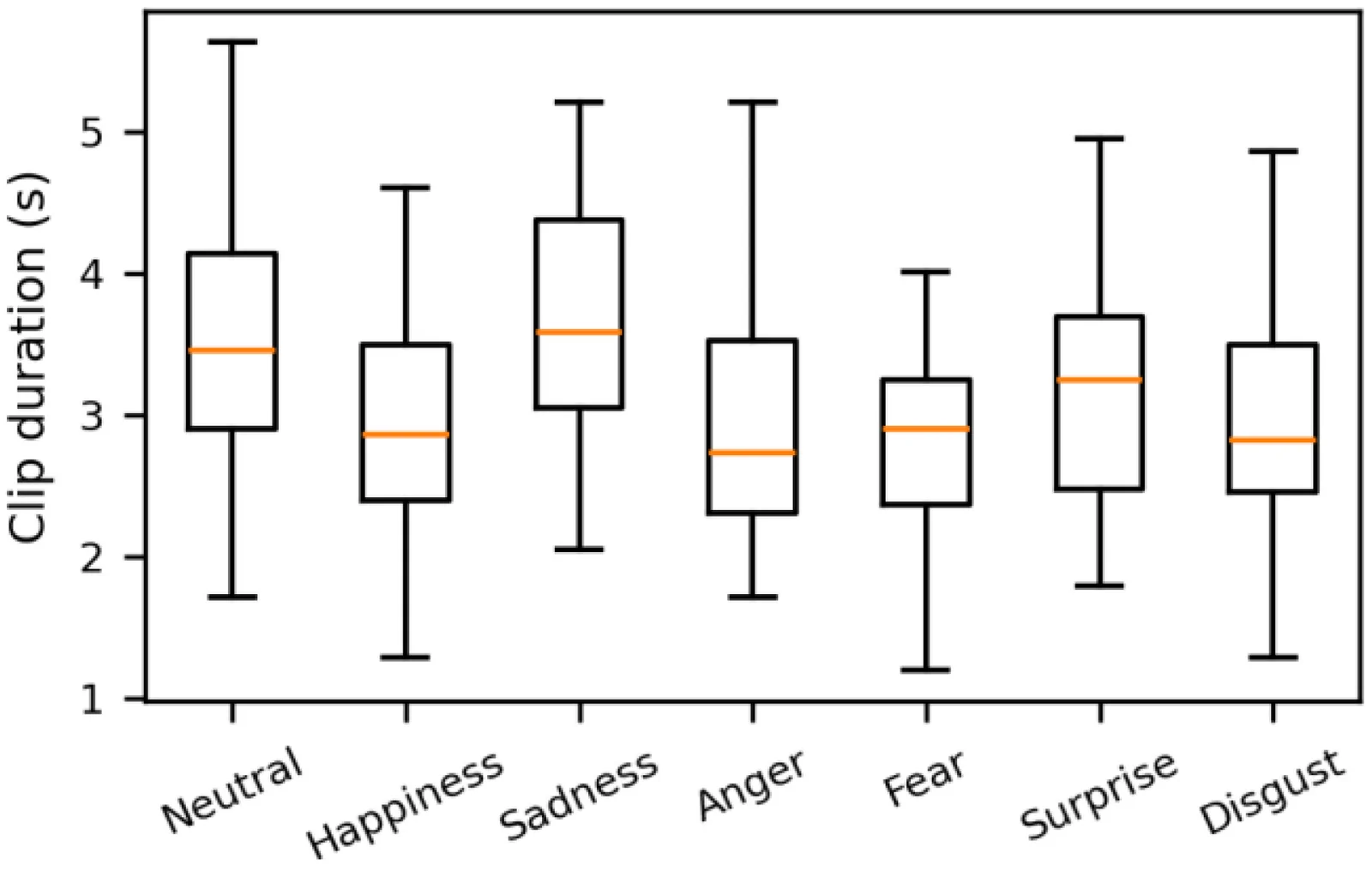
Distribution of clip durations by emotion (boxes: interquartile range and median). Whiskers exclude outliers beyond 1.5 × IQR; the full duration range is 1.2–10.1 s.

**Table 4:** Descriptive acoustics by emotion: mean clip duration, mean fundamental frequency (F0; Praat autocorrelation via parselmouth, pitch floor 75 Hz, ceiling 500 Hz, 40 ms analysis window advanced in 10 ms steps, averaged within a clip then across clips over voiced frames), and mean intensity (mean dB per clip) minus the same speaker's mean neutral intensity, so per-speaker recording gain cancels. Intensity uses Praat's Kaiser-20 window of 42.7 ms effective duration advanced in 10.7 ms steps. All four values are Praat's defaults for the 75 Hz floor. Each row averages the 60 clips of that emotion (10 speakers × 6 sentences); since every emotion is produced by the same ten speakers, the rows are comparable even though the F0 column pools female and male speakers. Descriptive statistics only, from scripts/acoustics_stats.py.

Emotion	Duration (s)	Mean F0 (Hz)	Intensity vs. neutral (dB)
Neutral	3.54	157	0.0 (ref.)
Happiness	2.91	221	+5.9
Sadness	3.74	154	−3.3
Anger	2.96	197	+8.8
Fear	2.81	214	+0.6
Surprise	3.09	202	+4.0
Disgust	3.02	156	+0.6

### 3.3 Accompanying files

Table 5 lists the released layout. The audio ships as recorded, raw WAV with no denoising or level normalization, so that users work with the same signals the listening test was run on. Two metadata files describe it. The first, corpus-sentences.json, holds the sentence and emotion inventory with codes, which maps the Albanian folder names to canonical English labels programmatically, and the second, manifest.csv, carries one row per recording (filename, speaker, intended emotion, sentence, duration, sample rate, bit depth, channels), so a subset can be selected, split, or filtered without reading the audio. speaker-demographics.csv supports speaker-independent splits and grouping by gender, age, or region.

**Table 5:** Contents of the archived deposit. The analysis scripts and the browser tools are distributed separately, in the code repository.

Path	Contents
recordings/spNN/	42 WAV per speaker (<emotion>/sentence_K.wav)
corpus-sentences.json	Sentence inventory and emotion codes
speaker-demographics.csv	Per-speaker metadata (Table 7)
manifest.csv	One row per recording (filename, speaker, emotion, sentence, duration, sample rate, bit depth, channels)
evaluations/	30 per-rater/speaker response CSVs plus aggregated results
consent-template	Blank bilingual consent form
LICENSE	CC-BY-4.0
checksums.md5	Integrity manifest

The perceptual-test responses are released as 30 CSV files, one per rater–speaker pair (audit_ev*NN*_sp*NN*.csv), with the fields in Table 6; every individual judgement is preserved, not only the aggregate, alongside the aggregated recognition rates, Fleiss’ κ, and integer confusion counts. The analysis scripts, distributed in the code repository, recompute those statistics, the acoustic summaries, and the machine baseline, and regenerate the figures reported here; the two browser tools there reproduce the collection protocol itself. A blank bilingual consent template, the licence, and MD5 checksums complete the deposit. No personally identifying information is distributed. Speakers and evaluators appear only as anonymized identifiers (sp*NN*, ev*NN*).

**Table 6:** Fields in each perceptual-test response file (audit_evNN_spNN.csv). One row per judgement, so the files join to the audio on the (speaker, emotion, sentence) key and preserve which rater gave which response.

Field	Description	Example
Rater	Anonymized evaluator ID	ev01
speaker	Anonymized speaker ID	sp02
sentence	Sentence index (1–6)	4
true_emotion	Intended emotion (Albanian marker)	trishtim
response	Emotion chosen by the rater	trishtim
Order	Presentation order in the test	17

### 3.4 Reuse

Because each recording’s emotion label is its parent directory, the corpus loads without a separate label file. For example, in Python:

**Table:** 

import soundfile as sf
from pathlib import Path
for f in Path("recordings").glob("sp*/*/sentence_*.wav"):
emotion = f.parent.name # Albanian marker,
# e.g. trishtim
audio, sr = sf.read(f) # sr = 48,000

The folder name is the Albanian emotion marker; corpus-sentences.json maps it to the canonical English label. The anonymized perceptual responses (Table 6) join to the audio on the (speaker, emotion, sentence) key, so users can weight training labels by listener agreement or study inter-listener variability.

### 3.5 Speakers

The ten speakers are native Albanian speakers, balanced by gender (five female, five male) and spanning ages from 18 to over 55 (Table 7). They come from seven regions across Albania, spanning both the northern (Gheg) and southern (Tosk) dialect areas. This describes the speakers’ regional origin, not dialectal variation in the recordings. They all recorded in Standard Albanian (*gjuha letrare*), so the audio is not dialectally diverse. Recording in the standard required no special accommodation, since *gjuha letrare*, standardized in 1972 and based largely on Tosk, is the ordinary register for formal and public speech, and speakers from across the regions of Albania use it [9,10]. All reported at least one additional language, most commonly English, and variously Italian, French, German, or Greek.

**Table 7:** Speaker demographics. All are native Albanian speakers who recorded in Standard Albanian; “Region” is the speaker's area of origin.

Speaker	Gender	Age	Region	Other languages
sp01	F	45–54	Tiranë	en, it, de
sp02	F	35–44	Dibër	en, it
sp03	M	45–54	Korçë	en
sp04	F	45–54	Patos	en, it, fr
sp05	M	35–44	Librazhd	en, it
sp06	M	18–24	Tiranë	en
sp07	M	35–44	Tiranë	en, fr
sp08	F	18–24	Tropojë	en
sp09	F	18–24	Skrapar	en, de
sp10	M	55+	Tiranë	en, el

The speakers are not professional actors; they are members of the University of Tirana community and their families. This follows the reasoning of EmoDB’s authors, who declined to require trained actors on the grounds that trained performers learn to express emotion in an exaggerated way [4]. What matters for the labels is that the portrayals are recognizable to listeners, which the perceptual validation establishes (the Perceptual validation section).

## 4 Experimental Design, Materials and Methods

### 4.1 Sentences and emotion set

The six carrier sentences (Table 3) are semantically neutral in Albanian, minimizing lexical emotion cues so that emotional differences are expressed primarily through vocal delivery. Candidate sentences were drafted to be every day, grammatically simple, and free of emotionally loaded words, regional markers, and proper names, with a mix of short and long lengths and wording that flows naturally when spoken aloud. Everyday sentences were used rather than nonsense material, which tends to elicit stereotyped overacting [4]. More than twenty candidates were drafted; a linguist (co-author) culled them to those in the most fluent and natural Albanian, and native-Albanian team members then rated the remainder for plausibility across all seven emotions, as was done for SUBESCO, where three linguistic experts chose ten sentences from twenty candidates [6]. The six with the highest agreement were kept, three short and three long.

The seven emotional styles (Table 2) are the six basic emotions of Ekman [2,3] and a neutral reference, the same set used by SUBESCO [6]. Disgust, which is difficult to convey and recognize from voice alone [6], was retained for completeness and its recognition rate is reported below.

### 4.2 Speaker recruitment and screening

Speakers were recruited from the University of Tirana community and their families. Each candidate was interviewed individually, given a detailed explanation of the task, and made a trial recording; those whose portrayals the authors judged clear and natural were invited to record the full set. The released corpus is therefore a screened set of speakers rather than an unselected sample. Screening for expressive clarity follows the preselection used for EmoDB, where ten performers were chosen from a set of respondents by judged naturalness and recognisability [4], and for SUBESCO, where candidates auditioned before a panel of experts who selected the final speakers on the recognisability of their portrayals [6].

### 4.3 Recording procedure

Each speaker recorded alone in a quiet office at the University of Tirana with a Blue Yeti cardioid USB microphone (side-address, ∼15–20 cm), the input gain set against the loudest emotion with headroom to avoid clipping. Recording used a purpose-built, fully offline browser tool (the recording tool in Fig. 4), written to make self-recording as simple as possible for people with no recording experience. It needs no installation, runs from a single web page, and presents one emotion and one sentence at a time with four controls, record, listen, save, and next. At the end the speaker downloads the whole session as a single archive. The tool saves one WAV file per utterance, so there is no continuous take, no spoken markers, and no segmentation step, and no recording can be cut at the wrong point.

Browser-side audio processing (echo cancellation, noise suppression, automatic gain control) was disabled, so no denoising, gain normalization, or speech enhancement was applied. Recording ran in Google Chrome, capturing 32-bit float PCM through the Web Audio API and writing 16-bit mono WAV in the browser.

All 420 files were checked for format consistency (48 kHz, 16-bit, mono), level, and background noise. No sample in the corpus reaches full scale, so no file clips, and the median peak level is −15.6 dBFS, confirming the intended headroom.

Background noise is low and consistent across speakers (Fig. 3). For scale, the VoiceBank–DEMAND speech-enhancement benchmark treats 17.5 dB as its cleanest test condition, the rest running down to 0 dB [11]; every speaker’s median clip in AlbEmo sits at least 7 dB above even that cleanest case. The quantity plotted is the difference between the 95th and 5th percentiles of 20 ms frame energies, so it measures how far the speech rises above the background within a single recording rather than a mixing-ratio SNR; the median background floor is −53 dBFS. No noise suppression or normalization was applied at any stage, so these are raw measurements. Clipping and noise are both computed by scripts/audio_qc.py.

**Fig. 3:**
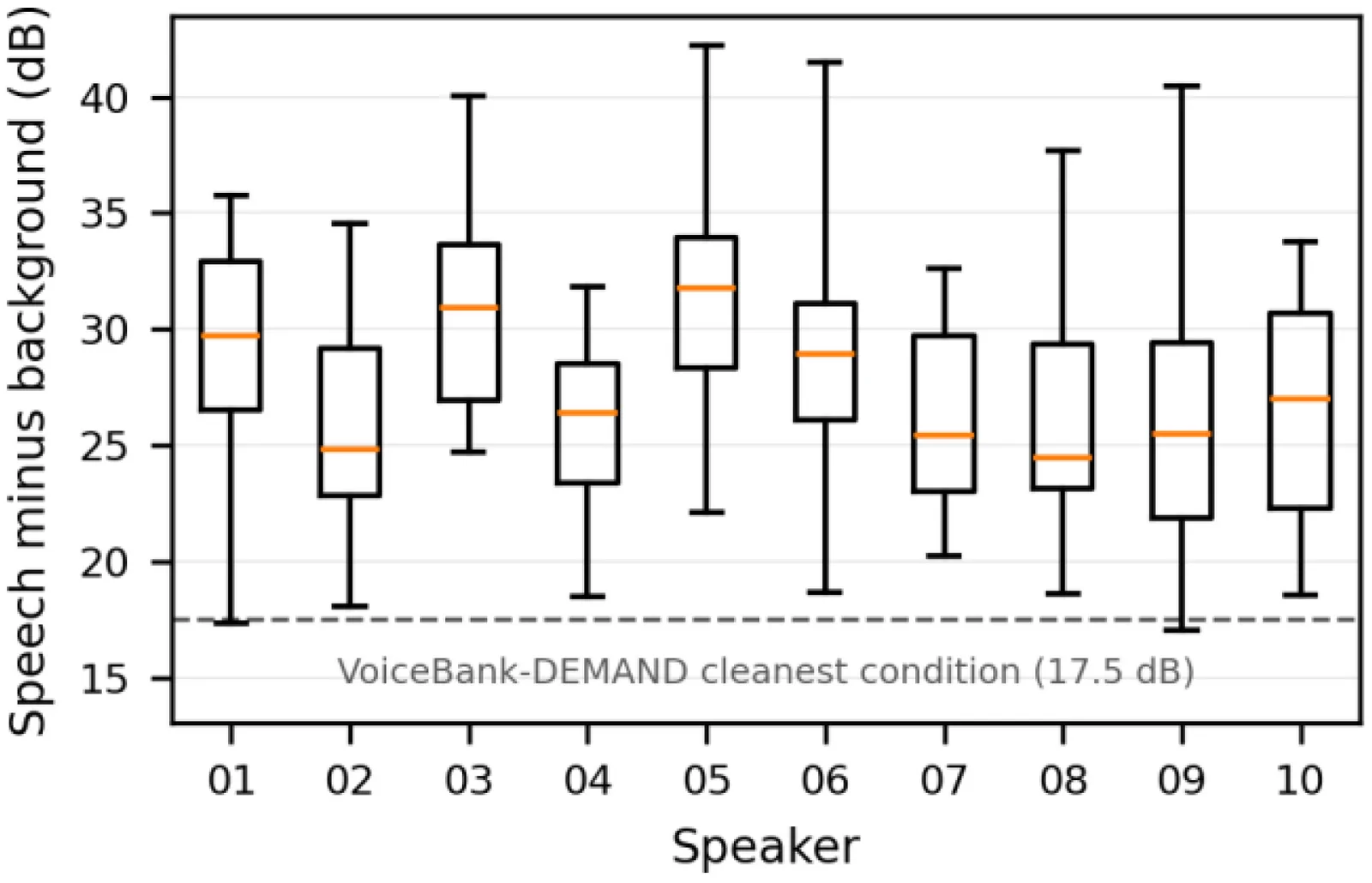
Level of the speech above the background noise floor, by speaker, over the 42 clips of each (boxes: interquartile range and median). Whiskers span the full range, unlike Fig. 2, so the bottom of each whisker is that speaker’s noisiest clip. Per-speaker medians run from 24.5 to 31.7 dB. The dashed line marks the cleanest test condition of the VoiceBank–DEMAND speech-enhancement benchmark [11], a scale reference rather than a target: only 2 of the 420 clips fall below it.

Emotions were recorded in the fixed order of Table 2. Before each emotion the tool displayed a short Albanian coaching cue asking the speaker to recall a real instance of the emotion and express it slightly more strongly than in everyday speech, the Stanislavski-recall approach used by EmoDB and SUBESCO. The cues are given in Table 8. Within each emotion the speaker read the six sentences, re-recording any line as often as they wished and keeping the take they were satisfied with. Recording privately and at one’s own pace was a deliberate design choice, because expressing emotion on neutral sentences is self-conscious work, and recording alone, with unlimited retakes and no one in the room, removes the inhibition of performing in front of an audience and lets speakers commit fully to the delivery. This matters all the more for speakers who are not trained performers. The authors explained the tool beforehand and were reachable throughout. The released audio is exactly what was recorded, and the perceptual validation reported below was performed on these same files.

**Table 8:** The Albanian coaching cue shown before each emotion block (Stanislavski-style recall), with an English gloss.

Emotion	Coaching cue (Albanian)	English gloss
Neutral	Ton normal, i qetë, pa emocion. Sikur po lexon një lajmërim.	Normal, calm tone, no emotion, as if reading an announcement.
Happiness	Kujto një çast që të ka bërë vërtet të lumtur. Buzëqesh ndërsa flet, zë i ngritur, i gjallë.	Recall a moment that truly made you happy; smile as you speak, raised, lively voice.
Sadness	Kujto një humbje ose mall. Ngadalëso, zë i ulët, i rënduar, sikur të ka ardhur keq.	Recall a loss or longing; slow down, low, heavy voice, as if grieved.
Anger	Kujto diçka që të ka zemëruar shumë. Fort, i prerë, i tensionuar, thuaje me inat.	Recall something that angered you; loud, clipped, tense, say it with rage.
Fear	Kujto një frikë reale. Zë i dridhur dhe i shpejtë, sikur je në rrezik.	Recall a real fear; trembling, fast voice, as if in danger.
Surprise	Sikur diçka e papritur sapo ndodhi. Zë i ngritur, i hapur, me habi.	As if something unexpected just happened; raised, open, astonished voice.
Disgust	Sikur ndjen diçka të neveritshme. Ton i shtrembëruar, me mospëlqim.	As if sensing something revolting; distorted tone, with dislike.

### 4.4 Perceptual validation

The emotional content was validated with a forced-choice listening test delivered through a second offline browser tool (the listening-test tool in Fig. 4). It is “blind” in that listeners saw only the audio and the seven category buttons, never the intended label or the file name. For each clip a native-Albanian listener chose one of the seven emotions; clips were presented in an independently shuffled order per evaluator, with replay allowed and no feedback. Four native-Albanian evaluators took part; three of them are also speakers in the corpus, but no one rated their own recordings, and every clip was judged by three of the four, for 1260 judgements over the 420 clips. We report, per emotion, the raw recognition rate (the share of a category’s clips that listeners labelled correctly) and the unbiased hit-rate of Wagner [12], which multiplies that share by the share of responses in the category that were correct, so a label a rater over-uses is penalized rather than rewarded. Writing $c_{ij}$ for the number of judgements in which a clip of intended emotion i received response j, with i and j ranging over the seven emotions, the raw recognition rate $H_{i}$ and the unbiased hit-rate $H_{u,i}$ of category i are

(1) $$H_{i}=\frac{c_{ii}}{\sum_{j}c_{ij}},\;H_{u,i}=\frac{c_{ii}}{\sum_{j}c_{ij}}\cdot \frac{c_{ii}}{\sum_{j}c_{ji}}$$

**Fig. 4:**
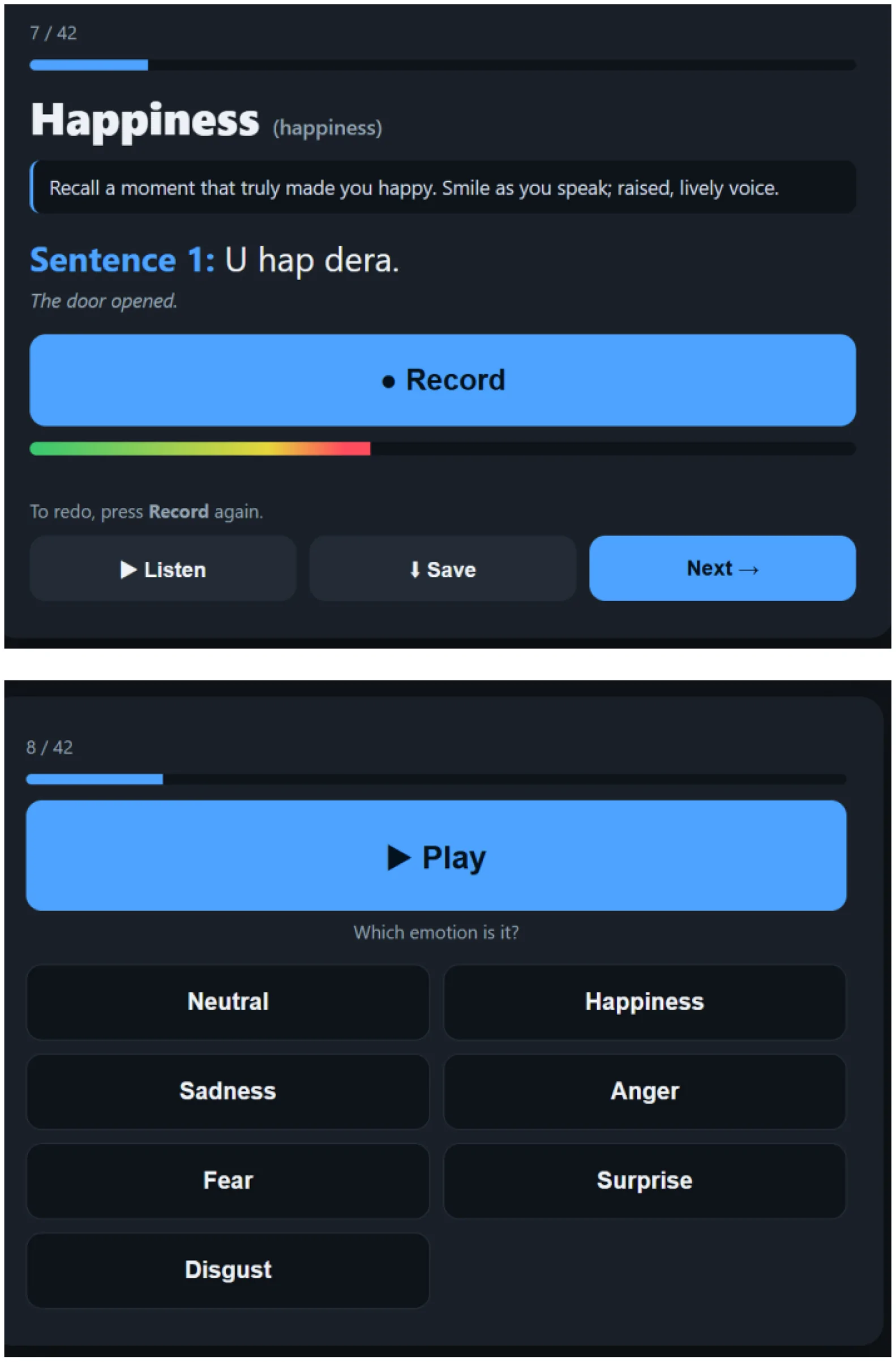
The two offline, browser-based data-collection tools (shown with English labels; participants used the Albanian versions, and the carrier sentence remains the Albanian stimulus). The self-recording tool presents one sentence and one emotion at a time with a coaching cue and saves one WAV per utterance; the blind forced-choice listening-test tool is used for perceptual validation.

Inter-rater agreement is quantified with Fleiss’ *κ* [13], computed over the N=420 clips, each rated by n=3 of the four evaluators into one of k=7 categories. Let $n_{ij}$ be the number of raters who assigned clip i to category j, so $\sum_{j}n_{ij}=n$. The proportion of all assignments made to category j, and the extent of agreement on clip i, are

(2) $$p_{j}=\frac{1}{Nn}\sum_{i=1}^{N}n_{ij},\;P_{i}=\frac{1}{n(n-1)}\left(\sum_{j=1}^{k}n_{ij}^{2}-n\right),$$

and *κ* compares the mean observed agreement $\bar{P}=\frac{1}{N}\sum_{i}P_{i}$ with the agreement ${\bar{P}}_{e}=\sum_{j}p_{j}^{2}$ expected if raters assigned categories at random with those same marginal proportions:

(3) $$\begin{array}{c} \kappa =\frac{\bar{P}-{\bar{P}}_{e}}{1-{\bar{P}}_{e}}. \end{array}$$

A κ of 0 means agreement no better than chance and 1 means complete agreement. Both measures are computed by scripts/score_audit.py. Every individual judgement is released, so agreement can be recomputed or reweighted.

Overall recognition was 73% (918/1260; chance =1/7≈14%), with a mean unbiased hit-rate of 54% and Fleiss’ κ=0.59, at the upper end of the “moderate” band of Landis and Koch [14]. This is in the range reported for comparable acted corpora. Over the identical seven emotions, SUBESCO reports 71% raw recognition, a 52% unbiased hit-rate, and κ=0.58 [6]; RAVDESS reports κ=0.53–0.61 across emotional intensities [5]. Fig. 5 gives the rates by emotion and Fig. 6 the rates by speaker, which range from 61% to 81% around a corpus mean of 73%; no speaker falls near chance, so the emotional signal is present throughout the corpus rather than carried by a few strong performers.

**Fig. 5:**
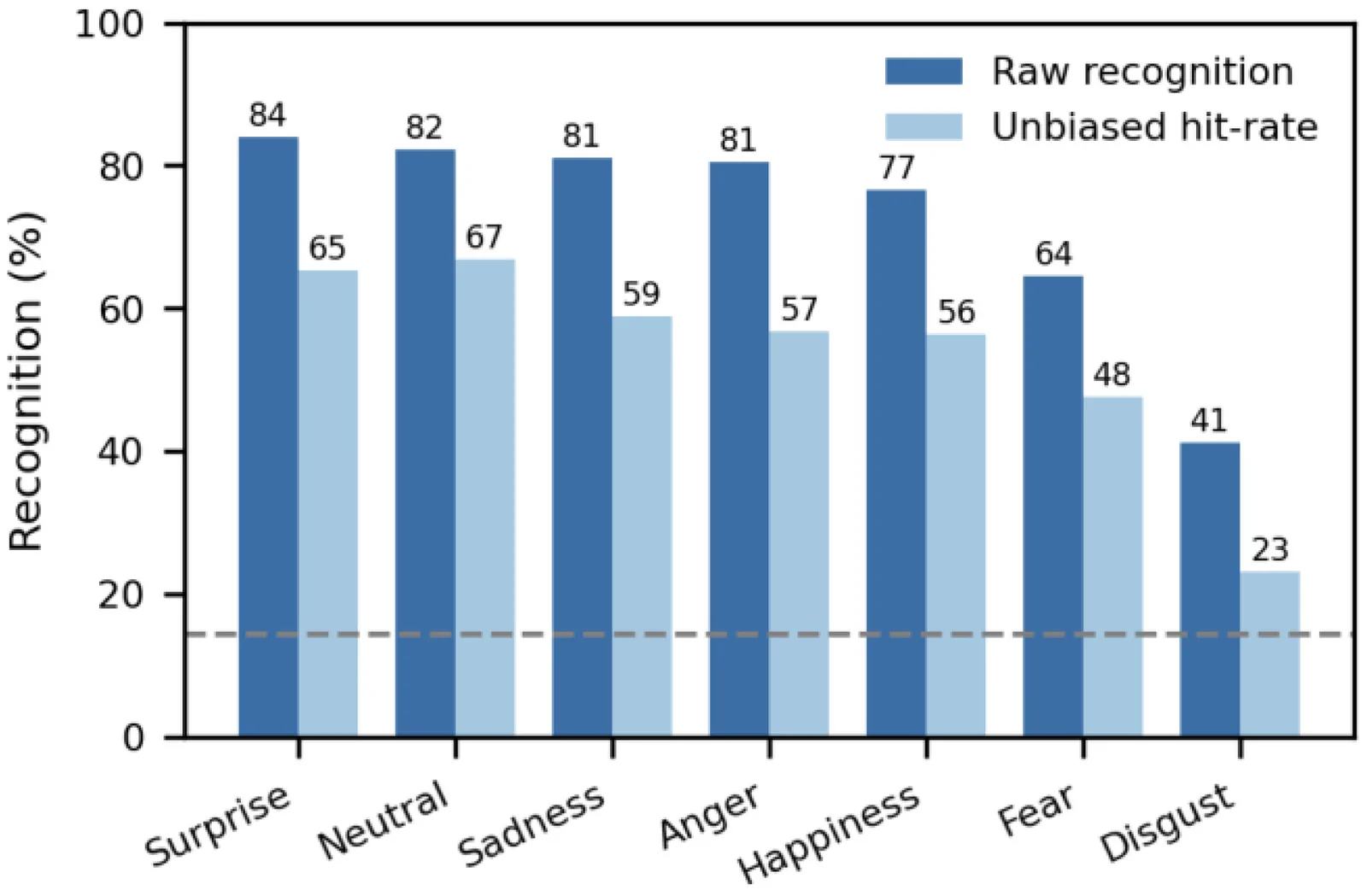
Per-emotion results of the listening test: raw recognition rate and unbiased hit-rate (Wagner), in percent, each over the 180 responses collected for that emotion (60 clips × 3 raters). Values are printed on the bars and the dashed line marks chance (≈14%).

**Fig. 6:**
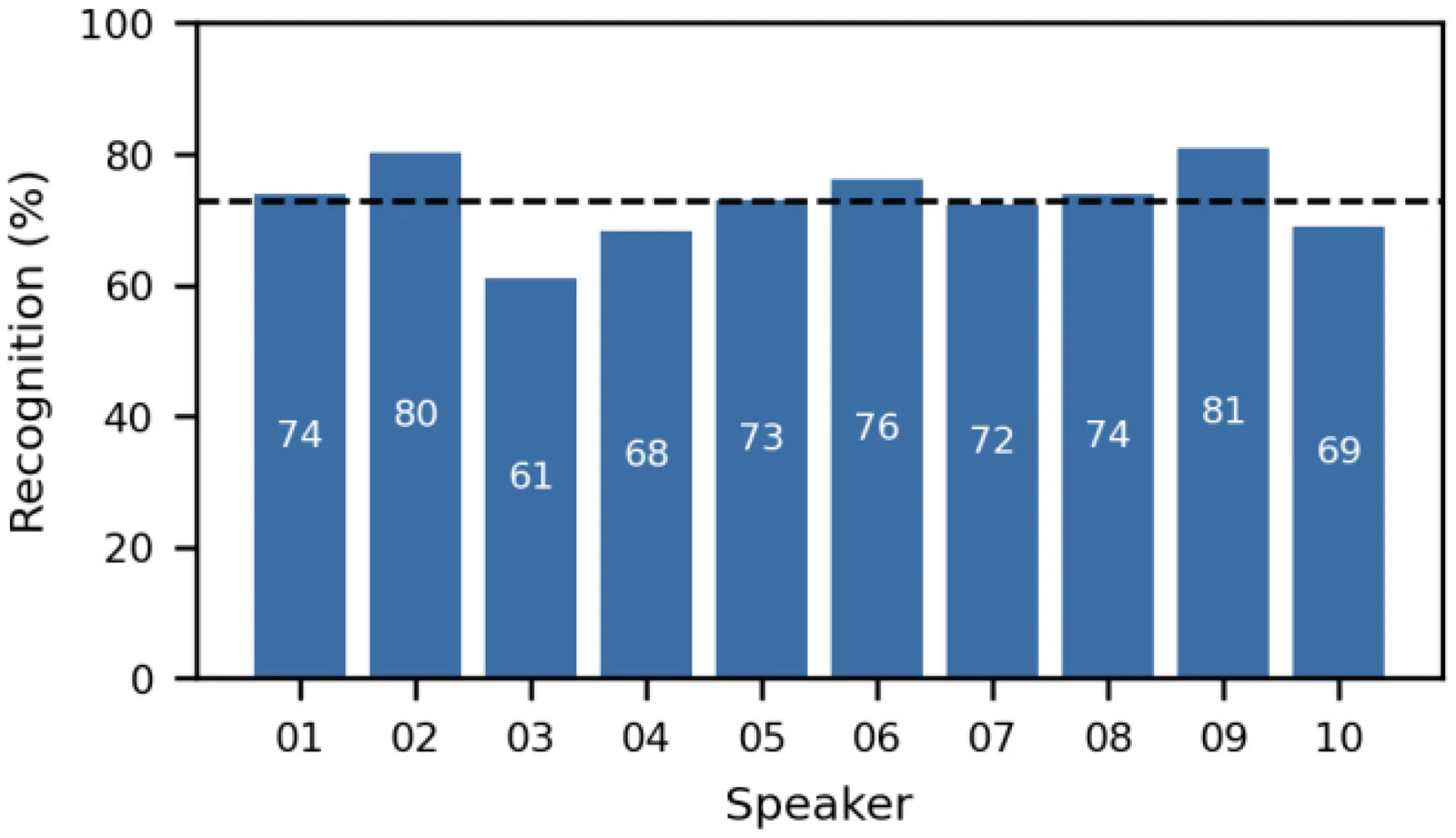
Recognition rate by speaker, each over the 126 responses collected for that speaker (42 clips × 3 raters). The dashed line marks the corpus mean of 73%.

The confusion matrix (Fig. 7) has a clear diagonal, in that for every emotion the intended category is the one listeners chose most often, from 84% for surprise down to 41% for disgust, in each case well above the 14% chance level. Outside disgust, no single confusion exceeds 9%. The errors that do occur follow familiar perceptual lines. Disgust is most often heard as anger (18% of its responses) or sadness (13%), the two largest off-diagonal entries in the matrix, and happiness and surprise are taken for each other in both directions (9% and 8%). The errors on fear, the second-weakest category, are spread across several emotions rather than concentrated in one. The same two categories are the hardest elsewhere. In SUBESCO’s seven-emotion phase, fear and disgust are likewise the two least recognized, with disgust the weakest of all, and its authors ran a second evaluation phase with disgust removed because of the confusions it introduced [6].

**Fig. 7:**
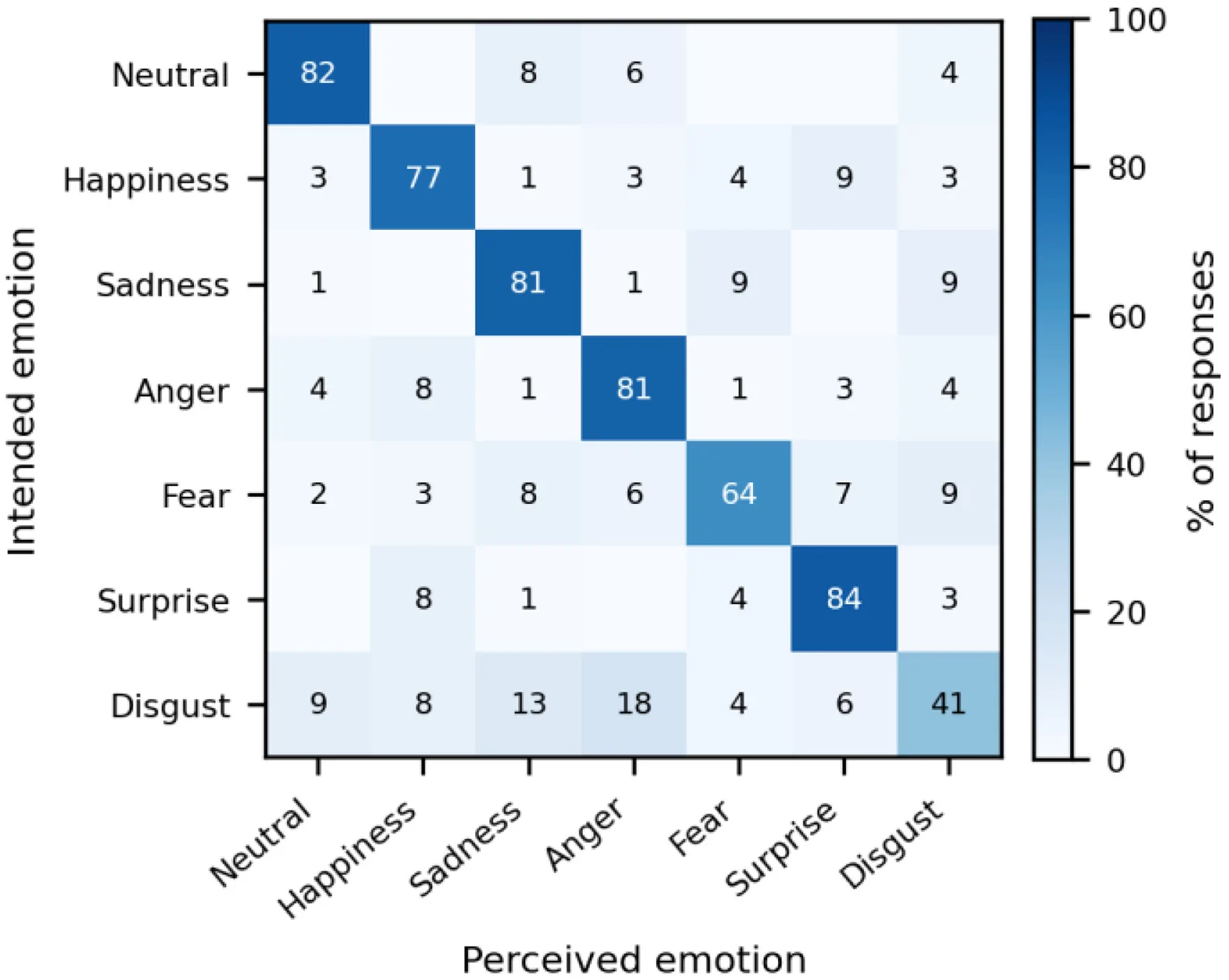
Human confusion matrix (row = intended, column = perceived emotion, row-normalized %, over the 180 responses per intended emotion; integer counts are released). Diagonal entries are the raw recognition rates.

### 4.5 Machine-readable baseline

As a single reference point for machine use, we report a self-supervised separability baseline built from frozen WavLM-base [15] embeddings, time-mean-pooled at 16 kHz, with a nearest-centroid classifier under cosine similarity, macro-averaged. The encoder layer is the one with the highest per-speaker silhouette, chosen by that same rule for every corpus rather than by accuracy; for AlbEmo it is layer 0. Per speaker, each of the 42 utterances is labelled by the nearest of that speaker’s own emotion centroids computed from the other 41 (leave-one-utterance-out), giving 74% average per-speaker accuracy over the seven emotions.

Comparing corpora requires a shared label set, so the figures that follow use the six emotions common to all three (EmoDB has no surprise category). On that six-way task the same pipeline gives 78% per speaker for AlbEmo, 77% for EmoDB [4], and 73% for RAVDESS [5]. Pooling the speakers within each corpus, and classifying each utterance against centroids built from the remaining pooled utterances, gives 51% for AlbEmo, 51% for RAVDESS, and 64% for EmoDB. These are pooled leave-one-utterance-out figures rather than speaker-independent ones, since a speaker’s other utterances remain in the training centroids, and every corpus falls once emotion representations have to be shared across speakers. The full procedure, applied identically to all three corpora, is in scripts/emo_separability_corpus.py. We report this only to document that the recordings carry recoverable emotional structure; a full modelling study is outside the scope of this data article.

## Limitations

Like EmoDB, RAVDESS, and SUBESCO, the emotions are portrayed rather than spontaneous, so results need not transfer directly to naturally occurring emotional speech. All recordings were made with one microphone in one room, a deliberate control that also means the corpus offers no channel or environment variability. Emotional intensity was neither varied nor annotated, and no time-aligned or phonetic transcription is provided beyond the sentence identity.

The perceptual validation was also carried out within a small pool. Three of the four evaluators are themselves speakers in the corpus, and both groups are drawn largely from the same university community. No one rated their own recordings, but familiarity with a talker is likely for at least some evaluator-speaker pairs, and we did not document which voices each evaluator knew. Familiarity may aid recognition, so the reported rates could be somewhat higher than listeners with no acquaintance with the speakers would produce.

## Ethics Statement

The study was approved by the Ethics Council of the University of Tirana, the institution implementing the project (Decision No. 85 of 16 June 2026; Rectorate protocol No. 1813/3 of 30 June 2026). All participants were adults, none belonging to a vulnerable group, and were paid an honorarium for their time. The research was carried out in accordance with the Declaration of Helsinki. Each gave written bilingual (Albanian and English) informed consent, with separate opt-ins for recording, for public release, and for demographic information, and was informed that voice is identifying and that, once distributed under CC-BY-4.0, released recordings cannot be recalled from downloaded copies. Signed consent originals are held securely at the University of Tirana and are not shared with third parties; only a blank consent-form template is distributed with the corpus. No personally identifying information appears in any file or filename; speakers and evaluators are anonymized (sp*NN*, ev*NN*).

## Credit Author Statement

**Emiranda Loka:** Investigation, Methodology, Project administration, Supervision, Validation, Writing – review & editing; **Ilma Lili:** Conceptualization, Investigation, Validation, Writing – review & editing; **Alda Kika:** Investigation, Methodology, Project administration; **Ana Ktona:** Investigation, Methodology, Project administration, Validation, Writing – review & editing; **Linda Mëniku:** Investigation, Methodology, Resources, Writing – review & editing; **Alex Thomo:** Conceptualization, Data curation, Formal analysis, Methodology, Project administration, Software, Supervision, Validation, Visualization, Writing – original draft.

## Declaration of Generative AI and AI-Assisted Technologies in the Manuscript Preparation Process

During the preparation of this work the authors used Claude (Anthropic) for assisting with experiment scripting and editing the manuscript. The authors reviewed and edited the output as needed and take full responsibility for the content of the published article.

## Data Availability

ZenodoAlbEmo: a perceptually validated emotional speech corpus for Albanian (Original data).
